# Experiences of digital physiotherapy during pregnancy and after childbirth: A qualitative study

**DOI:** 10.1016/j.invent.2024.100768

**Published:** 2024-08-22

**Authors:** Frida Johnson, Sara Frygner Holm, Andrea Hess Engström

**Affiliations:** Uppsala University, Department of Women's and Children's Health, Uppsala, Sweden

**Keywords:** Women's health, Physiotherapy, Qualitative research, Pelvic pain, Pelvic floor disorder, Digital health

## Abstract

**Background:**

Pelvic girdle pain, low back pain, and pelvic floor dysfunction can affect women's mobility, quality of life, and well-being during pregnancy and the postpartum period. Digital interventions for treating perinatal depression and lifestyle changes have been studied. Research on digital physiotherapy for musculoskeletal issues related to pregnancy and the postpartum period is sparse.

**Methods:**

This qualitative study involved in-depth, semi-structured interviews with 19 participants, of whom six were pregnant and 13 had given birth. Participants were recruited from a private clinic in Sweden through convenience sampling and had received digital physiotherapy prior to the interviews. An interview guide with questions exploring participants' experiences of digital physiotherapy, including its impact on musculoskeletal issues and daily life, and their motivation for seeking digital healthcare was used. Data were analyzed using a qualitative content analysis with an inductive approach.

**Results:**

The analysis resulted in two main categories: *Finding a new way into physiotherapy treatment* and *Personalized progress through tailored physiotherapy*. These main categories encompassed four generic categories: *Convenience and dissatisfaction motivators for digital physiotherapy*, *A dual experience – appreciated but not always comprehensive, Being involved in the rehabilitation process,* and *Perceived physical and mental improvements after digital physiotherapy*.

**Conclusion:**

Digital physiotherapy was well-accepted and perceived as beneficial for managing musculoskeletal symptoms during pregnancy and after childbirth. High accessibility and flexibility were considered advantages. However, inability to undergo a physical assessment was a challenge. Digital physiotherapy may be recommended as a complement to usual care, particularly for women with limited access to a physiotherapist specialized in women's health. Future studies exploring digital physiotherapy's efficacy for musculoskeletal issues during pregnancy and after childbirth are highly recommended.

## Highlights

• Flexibility and accessibility motivated seeking digital physiotherapy.
• Physiotherapists' expertise was highly regarded.
• A focus on information was valued highly.
• Inability to undergo physical assessment is a challenge with digital physiotherapy.
• Digital physiotherapy is accepted to manage pregnancy and postpartum issues.

## 1 Background

The journey of pregnancy and childbirth is a transformative experience, often accompanied by significant physical changes such as alterations in posture, weight distribution, and hormonal levels (Fiat et al., 2022). These changes can impose additional strain on muscles, ligaments, and joints, leading to symptoms such as pelvic girdle pain (PGP) and pelvic floor dysfunction (PFD) (Bozkurt et al., 2014; Fiat et al., 2022). Pregnancy-related physical changes encompass hormonal, biomechanical, and neurological adaptations during pregnancy, which may contribute to musculoskeletal problems during pregnancy (Fiat et al., 2022). Pelvic pain and pelvic floor dysfunction can affect women's mobility, quality of life, and well-being during pregnancy and the postpartum period (Elden et al., 2016; Robinson et al., 2018; VanWiel et al., 2024; Wang et al., 2022).

Pelvic girdle pain and low back pain (LBP) typically manifest in the second trimester of the pregnancy and may worsen as the pregnancy progresses (Casagrande et al., 2015; Fiat et al., 2022). They affect approximately 70–86 % of pregnant women and may limit daily activities such as walking, sitting, working, and performing daily tasks (Close et al., 2016; Gutke et al., 2018). Pelvic floor dysfunction is also a common issue during pregnancy and after childbirth, and can lead to urinary and fecal incontinence, bulging symptoms, and dyspareunia (Palmieri et al., 2022). Further, symptoms related to PFD have been associated with poor mental health after childbirth (VanWiel et al., 2024). Around 40 % of women report urinary incontinence during pregnancy (Moossdorff-Steinhauser et al., 2021a), and approximately one third experience incontinence 12 months after childbirth (Moossdorff-Steinhauser et al., 2021b). Dyspareunia has been reported by 40 % of women six months after a vaginal delivery (Lagaert et al., 2017).

Diagnosis is made based on medical history and physical assessment of muscles and joints (Casagrande et al., 2015; Grimes and Stratton, 2023). Traditional management typically involves physiotherapy interventions during pregnancy and after childbirth (Critchley, 2022; Liddle and Pennick, 2015). Individualized exercise can reduce pain, disability, and sick leave for patients with PGP and LBP during pregnancy (Liddle and Pennick, 2015). Similarly, tailored pelvic floor muscle training can prevent and treat urinary incontinence (Mørkved and Bø, 2014). Physiotherapy has also shown positive outcomes for dyspareunia, but its effect after childbirth is less studied (Fernández-Pérez et al., 2023).

There is growing evidence supporting digital health interventions for treating musculoskeletal pain conditions (Valentijn et al., 2022). During pregnancy and the postpartum period, many women rely on digital resources for information regarding their health and the health of their children (Slomian et al., 2017; Vogels-Broeke et al., 2022). Accessing physiotherapy services may be challenging due to a lack of knowledge concerning where to seek help after childbirth (Molin et al., 2022). While digital interventions have shown positive outcomes for treating perinatal depression, alcohol and tobacco cessation during pregnancy, and weight management postpartum, research on digital physiotherapy for musculoskeletal issues related to pregnancy and the postpartum period is sparser (Sherifali et al., 2017; Silang et al., 2021; Xie et al., 2024). Digital physiotherapy may increase access to healthcare regardless of geographical limitations and contribute positively to patient satisfaction among patients and physiotherapists, while reducing costs (Bennell et al., 2021; Nizeyimana et al., 2022). However, a gap remains in our understanding of how women experience digital physiotherapy for musculoskeletal problems related to pregnancy and after childbirth.

### 1.1 Aim

The aim of this study was to describe women's experiences of digital physiotherapy during pregnancy and after childbirth.

## 2 Methods

### 2.1 Study design and setting

This qualitative study was conducted in Sweden in 2023 and is reported in accordance with the Consolidated Criteria for Reporting Qualitative Research (COREQ) guidelines (Tong et al., 2007).

### 2.2 Participants and recruitment

The study population consisted of women who received digital physiotherapy treatment for musculoskeletal symptoms related to pregnancy and childbirth. Recruitment took place in 2023 using convenience sampling, which was considered complete based on information power principles (Malterud et al., 2016). During the recruitment period, 387 individuals were invited to participate, of whom 25 expressed interest, with 19 women included. Reasons for choosing not to participate were lack of time or were not specified.

Physiotherapists working at a private clinic in Stockholm, Sweden, sent out invitations via the digital healthcare clinic's chat function to all eligible patients they had treated in the preceding 12 months. Patients who were interested in participating received written information about the study prior to inclusion. Inclusion took place after signed informed consent was obtained.

The inclusion criteria were being over 18 years old, either being pregnant or having given birth within 12 months before inclusion, having undergone at least one digital physiotherapy session for musculoskeletal symptoms related to pregnancy or childbirth, and being able to communicate fluently in Swedish. Exclusion criteria were having received prior treatment from the interviewer and receiving face-to-face physiotherapy during the digital treatment period.

The median age of the participants was 32 years (interquartile range 31–35), and the majority (84 %) held a university degree. Participants resided in different geographical regions in Sweden. Six participants were pregnant and 13 had given birth in the preceding 12 months. A total of 15 % of the participants postpartum had received digital physiotherapy both during and after pregnancy. Among participants in the postpartum period, 85 % had had a vaginal birth and 15 % underwent cesarean section. Fifteen women sought digital physiotherapy for PGP and/or LBP, and four women for PFD. The participants reported varying levels of physical activity, from below the recommended minimum to high-intensity training. Further, 84 % of participants reported previous experience with digital healthcare.

### 2.3 Digital physiotherapy

Digital physiotherapy was delivered by a private healthcare provider based in Sweden. Treatment was provided by physiotherapists with experience in women's health. An application that enables video calls was used for consultations. Patients had access to a chat function, digital treatment programs, and articles about women's health within the application. Despite being a private clinic, all patients paid the same fee for a physiotherapy consultation as they would have in the public healthcare system, as the cost was subsidized by public funds. No referral was required for physiotherapy consultations. However, healthcare professionals, such as gynecologists and midwives, may also refer patients to a physiotherapist if deemed necessary. In this context, a referral means that a patient was advised to contact the clinic following a consultation with a gynecologist or a midwife.

The treatment consisted of an initial assessment session during which a thorough medical history was taken and an individualized treatment plan was established in collaboration with the patient. Treatment comprised patient education, pain management advice, and tailored exercise programs based on the patient's needs. Patients who received treatment recommendations were offered follow-up visits.

### 2.4 Data collection

Data were collected through in-depth semi-structured individual interviews conducted digitally via Uppsala University's account in Zoom, an online meeting platform. All interviews were audio-recorded for analysis, and access to the meeting link was restricted to the relevant participant. The interviewer ensured the security of the session by locking the meeting after the participant joined, preventing the participation of others during the meeting.

The interview guide consisted of open-ended questions exploring participants' experiences of digital physiotherapy, including its impact on musculoskeletal issues and daily life, and their motivation for seeking digital healthcare. Follow-up questions were used to encourage the participants to develop their statements. The length of the interviews ranged between 17 and 41 min (median = 28 min). One pilot interview was conducted to test the interview guide and resulted in the addition of one question on experiences of digital healthcare.

The interviews were performed by the first author (FJ) under supervision. FJ is a master student in physiotherapy who also worked at the clinic as a physiotherapist and manager. The interviewer has long professional experience in the field of women's health physiotherapy.

### 2.5 Data analysis

Data were analyzed using an inductive qualitative content analysis as described by Elo and Kyngäs (2008). All interviews were transcribed verbatim and read multiple times to gain a deeper understanding of the data. Data were organized by FJ through open coding, followed by categorization into subcategories. These subcategories were then grouped into generic categories, from which the main categories emerged through abstraction (Table 1). The entire analysis process was triangulated with two other researchers (SFH and AHE) through reflexive dialogue until a consensus was reached. AHE (PhD) is a specialized women's health physiotherapist, whereas SFH (PhD) is specialized in pediatric physiotherapy. Both have experience of qualitative research methods.

**Table 1:** Example of analysis process, from coding to building a main category.

Unit of analysis	Code	Subcategory	Generic category	Main category
It was beyond expectation, even if the physiotherapist could not physically assess me. She was very good at instructing me to do different exercises and to understand where I had pain.	Digital assessment beyond expectation	Flexibility and accessibility during treatment	A dual experience – appreciated but not always comprehensive	Finding a new way into physiotherapy treatment

### 2.6 Ethical considerations

The study received approval from the Swedish Regional Board of Ethics (registration number: 2022–06977-01, date: 2023-02-01), and was carried out in accordance with the General Data Protection Regulation (GDPR) and the Declaration of Helsinki. Given the potentially sensitive nature of the topics discussed, participants were informed about available healthcare support and contact information for the one of the researchers in the research team (SFH). However, none of the participants reported experiencing discomfort during the study.

## 3 Results

The findings of this study describe the experiences of digital physiotherapy during pregnancy and after childbirth, resulting in two main categories: *Finding a new way into physiotherapy treatment* and *Personalized progress through tailored physiotherapy*. These main categories encompass four generic categories: *Convenience and dissatisfaction motivators for digital physiotherapy, A dual experience – appreciated but not always comprehensive, Being involved in the rehabilitation process,* and *Perceived physical and mental improvements after digital physiotherapy* (Table 2).

**Table 2:** Description of subcategories, generic categories, and main categories.

Subcategories	Generic categories	Main categories
Being dissatisfied with regular care Meeting a physiotherapist with the right expertise Time-saving and flexible visits Positive experiences of digital healthcare drive digital adoption	Convenience and dissatisfaction motivators for digital physiotherapy	Finding a new way into physiotherapy treatment
Benefits and challenges of physical assessments and understanding exercises Flexibility and accessibility during treatment Collaboration between digital and usual care desirable	A dual experience – appreciated but not always comprehensive	
Enhancing recovery through individualized physiotherapy Participating in and feeling seen in treatment Valuing continuity of care and structured rehabilitation plan	Being involved in the rehabilitation process	Personalized progress through tailored physiotherapy
Experiencing improvements in symptoms Experiencing improved well-being	Perceived physical and mental improvements after digital physiotherapy	

### 3.1 Main category: finding a new way into physiotherapy treatment

This main category encompassed participants' descriptions of the factors that motivated them to seek digital physiotherapy, as well as the challenges and advantages associated with this mode of treatment.

#### 3.1.1 Generic category: convenience and dissatisfaction motivators for digital physiotherapy

Dissatisfaction with usual care both during and particularly after pregnancy and getting to consult a physiotherapist with expertise in women's health were reported as motivators for seeking digital care. The participants described a general lack of knowledge regarding musculoskeletal pregnancy-related issues among healthcare providers, prompting them to search for information online.

The participants described feelings of loneliness, concerns that their issues were not serious enough to justify burdening the healthcare system, and difficulties in navigating complex referral processes, characterized by inefficient communication between healthcare instances. As one participant put it: *“It's been a healthcare carousel since then. Yeah, I was sent around [...] care in women's health hasn't been all that great. [...] No one really knew where I was supposed to turn, but then, after a lot of back and forth, I finally got an appointment.”* (P9)*.*

The convenience of accessing physiotherapy services from home, particularly when experiencing pain and fatigue, along with the possibility to get appointments more quickly than in usual care were also motivators for seeking digital physiotherapy. Not needing to take time off work, less exposure to viral infections, and the ability to attend sessions with the baby lowered the barriers for seeking digital care. As one participant put it: *“It's easier to get 20 minutes when the baby is sleeping, than to also get the time to go to the hospital or wherever you have to go, then have the visit and then get back home.”* (P12)*.*

Participants with previous experience of pregnancies and physiotherapy found it easier to seek and benefit from digital physiotherapy. They viewed digital services as a natural extension of daily life.

#### 3.1.2 Generic category: a dual experience – appreciated but not always comprehensive

The participants highlighted both advantages and challenges associated with receiving physiotherapy digitally. Some participants expressed a wish for physical examinations or manual treatments. As one participant said: *“I can imagine that maybe you would like a physical assessment after the birth. Mainly the abdominal muscles, because I don't know how divided they were before and what they look like now.”* (P13).

The participants found it easier to comprehend the recommended exercises through video than using traditional paper-based training programs. They valued the opportunity to ask questions and receive feedback on their performance: *“And she could show or describe where I was having pain on my body, and she could show the exercises. And then she wanted me to do the exercises so she would see that I did them right. Yeah, I thought it worked great.” (P16).* However, some participants encountered difficulties in understanding the recommended treatment during the digital visits and wished for feedback on the exercises performed. Instructions on pelvic muscle training were reported as being challenging to grasp, regardless of whether the visit was conducted digitally or face-to-face.

The user-friendly digital formats, flexibility of treatment, and time-saving were particularly valued after treatment had been initiated. The participants appreciated having access to a chat function with the physiotherapist between visits, as well as the ability to access treatment recommendations online. As one participant stated: “*(…) you have this chat function and you have the training program collected in one place, so in that way it is easy to keep everything together: the healing process or, like, the case and the follow-up. You have the information collected and can also see what you've written before, and if you don't remember when you contacted them, so it's very easy to get an overview of the treatment.*” (P9). Participants expressed a wish for reminders and encouragement to continue with the recommended treatment, to enhance their motivation.

The participants emphasized the importance of collaboration among healthcare professionals and suggested that a combination of digital and face-to-face physiotherapy with one and the same physiotherapist would be ideal for addressing their concerns. Face-to-face visits were considered more appropriate when digital sessions did not offer sufficient assistance, particularly for more complex issues or as a complement to usual care. Digital visits could feel more impersonal and be perceived as insufficient when an assessment of the pelvic floor muscles or abdominal muscles was needed.

### 3.2 Main category: personalized progress through tailored physiotherapy

The main category *Personalized progress through tailored physiotherapy* included participants' descriptions of their involvement in the rehabilitation process and how they perceived changes in their well-being following digital physiotherapy.

#### 3.2.1 Generic category: being involved in the rehabilitation process

The participants described how digital physiotherapy lowered barriers to adhering to treatment recommendations and resuming exercise after childbirth and highlighted the importance of receiving tailored physiotherapy. As one participant put it: *“I felt already after the first time that it felt good and that the exercises that had been given were what was needed for me to get where I wanted.”* (P6).

Being satisfied with digitally delivered physiotherapy and feeling well-informed and involved in the rehabilitation process were also reported. The participants reported feeling that digital physiotherapy allowed for more focus on information and increased attention from the physiotherapist. As one participant explained: “*You get the full focus, there is no window or other people around. It's like we're sitting now, (…), especially when you have headphones, it is hard to focus on anything else. You get the full focus*.” (P13).

The participants appreciated the physiotherapist's supportive role, particularly when feeling anxious or having pain. One participant stated: *“Not only exercises, but also how you can think and work mentally. Because it affects a lot [...] that you're in pain and that you can't do what you usually do and then you feel insufficient and bad. She's also helped me work on the mental parts.”* (P11).

Continuity of care with one and the same physiotherapist throughout treatment, along with having a structured rehabilitation plan, was also perceived positively.

#### 3.2.2 Generic category: perceived physical and mental improvements after digital physiotherapy

The participants reported reduced pain and improved function, which enabled them to walk longer distances, perform everyday activities, and return to sports. *“Yeah, I can manage life now [...] I couldn't do anything before, not take care of my child or myself the way I wanted. So there's been a huge difference. Everyday life works and I think that's the most important thing, even if I need to adapt some things, [...] I can do everything today that I couldn't do before my treatment.”* (P14). Some participants also noted improvements in urinary and fecal leakage and credited digital physiotherapy for facilitating communication around these issues.

Increased confidence in the ability to perform activities of daily living and improvements in overall well-being, mood, and quality of life were also described. As one participant put it: *“I became happier and more positive again. And it's quite a large number of weeks, waiting twenty weeks for a birth and not being able to do anything. Going from first having my great joy in life taken from me, to now, like, helping me to be able to do it my way instead. So, it was quite a big difference for me mentally, that everything hasn't disappeared, I can do something.”* (P17). A few participants treated for PGP during pregnancy did not experience physical improvements until after childbirth, but said that the treatment helped them to cope better with everyday life.

## 4 Discussion

This study describes women's experiences of digital physiotherapy during pregnancy and after childbirth. Two main categories emerged: *Finding a new way into physiotherapy treatment* and *Personalized progress through tailored physiotherapy*. Motivators for seeking digital physiotherapy included dissatisfaction with usual care, flexibility, getting easy access to a physiotherapist, and receiving treatment from a physiotherapist with expertise in women's health. Although participants found digital assessment and treatment feasible, challenges such as not being able to undergo a physical examination were reported. Nonetheless, participants emphasized the importance of tailored physiotherapy, treatment continuity, and active involvement in their rehabilitation. Overall, the participants perceived that digital physiotherapy was helpful to manage musculoskeletal symptoms during pregnancy and after childbirth.

In the generic category “Convenience and dissatisfaction motivators for digital physiotherapy,” lack of information and difficulties in accessing usual healthcare were found to be reasons to opt for digital physiotherapy. Postpartum care routines may need adjustment, as many women start experience issues related to childbirth several months after the regular follow-up (Sultan and Carvalho, 2021). Difficulties in finding adequate information after childbirth can affect emotional and psychological well-being, suggesting a need for tailored information protocols for women during pregnancy and in the postpartum period (Molin et al., 2022).

Key motivators for digital physiotherapy included availability, convenience, and a positive attitude towards digital treatment, aligning with previous research on digital physiotherapy for musculoskeletal conditions (Bennell et al., 2021). In the present study, the participants expressed a desire to avoiding travel to clinics, highlighting that the preference for digital physiotherapy primarily stems from a perception of lack of support within usual healthcare, rather than from the digital format itself. Nonetheless, digital physiotherapy may be particularly suitable for patients requiring greater flexibility during treatment and may be especially relevant for those living in rural areas (Harkey et al., 2020).

In the generic category “A dual experience – appreciated but not always comprehensive,” the user-friendly technology, online assessment, and treatment process were highlighted. Previous research has found, similarly, that quality and usability impacted patients' usage and engagement in digital healthcare (O'Connor et al., 2016). However, some participants in our study expressed a wish for physical examination or manual treatment. In previous research, digital physiotherapy was perceived as inferior to face-to-face physiotherapy and challenges regarding assessments and manual treatment were described (Barton et al., 2022). Thus, although digital physiotherapy appears feasible and acceptable, it may be less suitable for patients requiring assessments that can only be carried out during a physical visit, for instance of pelvic floor muscle function.

Understanding the recommended treatment was described as straightforward. In a previous study, patients perceived that healthcare providers fully understood their health concerns and that clinicians were interested in them as human beings (Rose et al., 2021). Further, patients tend to be comfortable and empowered in their home environment (Hinman et al., 2017). It is possible that the relationship between the physiotherapist and patient during the digital visits facilitated focused communication and understanding of the information. Associations between factors affecting patient experiences and treatment outcomes are beyond the scope of the present study but should be further investigated in future studies.

Understanding pelvic floor muscle exercise instructions was challenging, regardless of visit type. Difficulties in correctly contracting the pelvic floor during physical assessments are common (Vermandel et al., 2015). Clear instructions and knowledge about the pelvic floor anatomy contribute to understanding and promote correct exercise performance (Ben Ami and Dar, 2018; Vermandel et al., 2015), including when delivered through video guidance (Kamalı et al., 2023), highlighting the importance of patient education and personalized care.

In the category “Participation in the rehabilitation process,” tailored physiotherapy was found to be considered important in promoting adherence to treatment. Deeper understanding of patient conditions enables patient-centered treatment (Wijma et al., 2017). Nevertheless, it cannot be excluded that musculoskeletal issues during pregnancy and after childbirth are more easily addressed by physiotherapists with expertise in women's health, affecting the experience of being involved in the rehabilitation process.

Lastly, in the category “Perceived physical and mental improvements after digital physiotherapy,” the participants reported improvements in physical and mental well-being. High adherence to pelvic floor muscle training with frequent follow-ups can prevent and treat urinary incontinence before and after pregnancy (Mørkved and Bø, 2014), but the current evidence of effects from exercise on pain and disability due to pregnancy-related LBP and PGP is limited (Liddle and Pennick, 2015). It is worth noting that the participants in the present study reported complaints of varying extent. Further studies on digital physiotherapy should prioritize identifying effects of treatment and the patient profiles that most benefit from digital physiotherapy, so as to enhance treatment recommendations.

### 4.1 Clinical implications

Digital physiotherapy appears to be well-accepted and was described as helpful in managing symptoms among women with musculoskeletal issues during pregnancy and after childbirth. For some patients, face-to-face treatment or combining digital and face-to-face physiotherapy may be ideal. Nonetheless, digital physiotherapy may allow real-time adjustments and accommodate the dynamic nature of pregnancy and the postpartum period. Currently, digital physiotherapy may be recommended as a complement to standard care. Physiotherapists educated in women's health seem to play a role in experiences of digital care, but the availability of such resources may vary in clinical practice.

### 4.2 Strengths and limitations

This is, to our knowledge, the first qualitative study that describes experiences of digital physiotherapy for pregnancy-related musculoskeletal problems, which can contribute to the development of future studies on digital physiotherapy.

A strength of the present study was the analysis of a rich material, with participants living in different geographical locations in Sweden and with different experiences of digital healthcare. This contributed to capturing a variation in experiences and descriptions of digital physiotherapy.

The recruitment took place through convenience sampling (Elfil and Negida, 2017), and it cannot be excluded that individuals who were satisfied with digital physiotherapy reported interest in participating in the study. There is also a risk that the participants held back some criticism as they were aware of the interviewer's position at the clinic.

The transcripts were not returned to the participants for recognition and feedback, although this could have strengthened the analysis process. Another limitation relates to the fact that the interviewer worked as a physiotherapist and manager of the clinic where the participants were recruited. However, none of the participants had contact with the interviewer prior to the study. Further, the analysis process was performed with conscious and self-critical reflection (Fleming, 2018), and triangulation was used to strengthen the study's credibility (Shenton, 2004).

The majority of the participants were highly educated, which is in line with previous studies on the usage of digital healthcare (Ratcliff et al., 2021). Thus, the results from the present study may be transferable to highly educated women in Sweden. Future studies should focus on women with other demographic backgrounds.

### 4.3 Conclusion

Digital physiotherapy was well-accepted and perceived as beneficial for managing musculoskeletal symptoms during pregnancy and after childbirth. Good accessibility and flexibility were considered advantages. However, not being able to undergo a physical assessment was a challenge. Digital physiotherapy may be recommended as a complement to usual care, particularly for women with limited access to a physiotherapist with specialist knowledge in women's health. Future studies exploring digital physiotherapy's efficacy for musculoskeletal issues during pregnancy and after childbirth are highly recommended.

## Author agreement

The manuscript is the authors' original work and has not been published previously, nor has it been sent for publication elsewhere. All authors have seen and approved the final version of the manuscript being submitted.

## CRediT authorship contribution statement

**FJ**: Conceptualization, formal analysis, writing original draft, writing – review and editing, visualization, funding Acquisition; **SFH**: Conceptualization, methodology, formal analysis, writing – review and editing, supervision; **AHE:** Methodology, formal analysis, writing original draft, writing – review and editing, visualization, supervision, project administration.

## Declaration of competing interest

The authors declare the following financial interests/personal relationships which may be considered as potential competing interests: Frida Johnson reports financial support was provided by Her Company AB. Frida Johnson reports a relationship with Her Company AB that includes: board membership, employment, and equity or stocks. The other authors (SFH and AHE) declare that they have no known competing financial interests or personal relationships that could have appeared to influence the work reported in this paper.
